# A Short Media Training Session Is Effective in Reinforcing Psychiatrists’ Communication Skills About Suicide

**DOI:** 10.3389/fpsyg.2021.733691

**Published:** 2021-09-16

**Authors:** Karl Walter, Marielle Wathelet, Sacha Valdenaire, Pierre Grandgenèvre, Nathalie Pauwels, Guillaume Vaiva, Charles-Edouard Notredame

**Affiliations:** ^1^Psychiatry Department, CHU Lille, Lille, France; ^2^Papageno Program, Lille, France; ^3^Fédération Régionale de Recherche en Psychiatrie et Santé Mentale Hauts-de-France (F2RSM Psy), Saint-André, France; ^4^Centre National de Ressources et Résilience pour les Psychotraumatismes (Cn2r), Lille, France; ^5^PSY Lab, Lille Neuroscience & Cognition Centre, INSERM U1172, University of Lille, Lille, France

**Keywords:** suicide, media, Werther effect, Papageno effect, psychiatrist, training

## Abstract

Because it has been associated with significant increases [through the Werther Effect (WE)] or decreases [through the Papageno Effect (PE)] of suicide rates, media coverage of suicide-related events is recognized as a prevention leverage. Unfortunately, the recommendations that the World Health Organization (WHO) has published to help journalists reporting on suicide remain poorly applied. The Mini Media Training (MMT) is a short media training session designed to increase psychiatrists’ ability to communicate about suicide during interviews. We aimed at assessing the effect of the MMT on psychiatrists’ ability to help journalists complying with the WHO recommendations. From June 2017 to December 2019, 173 physicians and residents in psychiatry were recruited during French national congresses. At baseline (T0) and 1 and 3 months later (T1), participants received the MMT, which consisted in a simulated interview where they we asked to answer a journalist about a mock suicide. Communication skills were measured with a score summing the number of delivered pieces of advice in relation to the WHO recommendations, with a maximum score of 33. A weighted score was also derived based on the degree of directivity needed for the participant to provide these items, again with a possible maximum of 33. A total of 132 psychiatrists participated in the study at T0 and T1. Both the weighted and unweighted score significantly increased from T0 to T1 (*d* = +2.08, *p* < 0.001, and *d* = +1.24, *p* < 0.001, respectively). Having a history of contacts with journalists, a short professional experience (<3 years) and prior knowledge of the WE, PE, and WHO recommendations were significantly associated with greater unweighted and weighted scores at baseline. The latter two variables also predicted greater T0–T1 improvement of the weighted score. These results suggest that the MMT could be effective for improving the ability of psychiatrists to guide journalists toward more responsible media coverage of suicide. As a short, easy to implement educational activity, the MMT could therefore be considered in association with other measures to help media professionals mitigating the WE and promoting the PE.

## Introduction

Despite sustained prevention efforts, suicide still accounts for 1.4% of premature deaths worldwide (Bachmann, 2018). In most Asian, American and European regions, it remains one of the top ten causes of age standardized years of life loss (Naghavi, 2019). In France, suicide has caused about 9,280 evitable deaths in 2016 (Observatoire National du Suicide, 2019).

In response to this alarming observation, the World Health Organization (WHO) has set, in its Global Mental Health Action Plan, the objective of a 10% decrease of suicides over the period 2013–2020 (WHO, 2019). Among prevention actions recommended as efficient, the WHO promotes the empowerment of media professionals in reporting on suicide-related events. For more than half a century, the literature has indeed provided robust evidence that incautious media coverage of suicide is associated with a substantial increase of suicide rates, especially in young populations (Phillips, 1974; Leon et al., 2014) and/or when a celebrity in involved (Niederkrotenthaler et al., 2020). Detrimental consequences of suicide-related reports have been tagged “Werther Effect” (WE) in reference to the European suicide epidemy that followed the publication of Goethes’ *Sorrow of the Young Werther* (Von Goethe, 1774). Conversely, more limited but growing evidence exist that specific media productions, such as testimonies of individuals who successfully coped with a suicidal crisis, could reduce suicidal ideations, increase life satisfaction, provide knowledge about suicide-related matters and promote help-seeking behaviors in readers or viewers (Niederkrotenthaler et al., 2019). Niederkrotenthaler et al. (2010) coined Papageno Effect (PE) the possible protective effects of such portray in reference to the resilient character of Mozart’s opera *The Magic Flute*.

Importantly, both the WE and PE have been related to specific characteristics of media productions. From a quantitative point of view, the risk of WE increases with the number and length of articles and broadcasts according to a dose-effect relationship (Etzersdorfer et al., 2004; Pirkis et al., 2006). From a qualitative point of view, using a sensational tone, describing with precision the mean and place of the suicidal event or displaying the term “suicide” in the headline have been related to stronger WE, presumably due to greater salience and emotional impact (Etzersdorfer et al., 2004; Pirkis et al., 2006; Niederkrotenthaler and Sonneck, 2007; Niederkrotenthaler et al., 2010; Notredame et al., 2015). Conversely, the literature suggests that suicide-related reports that contribute to debunk stereotypes around suicide and highlight help resources promote the PE (Niederkrotenthaler et al., 2014; Notredame et al., 2016).

To improve media coverage of suicide, the WHO published in 2008 eleven recommendations for media professionals (World Health Organization (WHO), and International Association for Suicide Prevention (IASP), 2008). Basically, the document encourages journalists to raise awareness about suicide without spreading misconceptions, to carefully consider the relevance of covering a suicide-related event that has already been reported, to avoid sensationalizing or normalizing style and wording, to remain vague when describing the mean and place of the suicide and to pay special attention when dealing with a celebrity suicide. In 2017, the WHO added a twelfth recommendation dedicated to the promotion of the PE by encouraging journalists to provide precisions about how to deal with stressors or suicidal thoughts and how to get help (World Health Organization (WHO), and International Association for Suicide Prevention (IASP), 2017).

Regrettably, release of national adaptations of the WHO recommendations has led to mixed results (Tatum et al., 2010; Chandra et al., 2014). Michel et al. (2000) for instance, observed that the publication of the Swiss guidelines resulted in an overall improvement of the quality of the reporting, but also in a significant increase of the number of articles dealing with suicide. This observation violates one of the most critical recommendation about avoiding redundancy in suicide-related information (Michel et al., 2000). Fu and Yip (2009) failed to find any significant difference in the compliance of media productions to the WHO recommendations before and after their national release. Likewise, Tatum et al. (2010) observed that the newspapers articles about suicide published in the 2 years after the release of the United States media guidelines did not consistently reflect their influence.

One possible account for the poor compliance of media productions with guidelines (Collings and Kemp, 2010) is that journalists are rarely aware of their responsibility about suicide and remain mostly uninformed of the existence of any recommendations (Jamieson et al., 2003). Therefore, rather than a simple publication and distribution process, a global interdisciplinary approach involving consultation and collaboration with media professionals may be needed for the WHO guidelines to be applied (Notredame et al., 2016).

In France, the Papageno Program works since 2015 at preventing suicide contagion and promoting access to care by leveraging different communication channels.^1^ One of its core goal consists in reaching better compliance with the WHO recommendations in media productions. Inspired by similar national initiatives (Skehan et al., 2009; Scherr et al., 2019; Duncan and Luce, 2020), the Papageno program carries out a multimodal strategy associating the spread of a French translation of the WHO guidelines with the publication of resources and the organization of training sessions for media actors, professionals and students. More recently, the program also considered strengthening the role of psychiatrists in the prevention of the WE and promotion of the PE.

Because they are frequently interviewed as experts about suicidal behaviors, psychiatrists are key resources to raise media stakeholders’ awareness about the possible consequences of suicide coverage and to help them addressing themes in relations with suicidal behaviors. However, psychiatrists and psychotherapists were shown to endorse very heterogenous theories and understandings about media effects on mental health (Arendt and Scherr, 2017). Most French medical curricula don’t include any course or training about public communication. Thereby, psychiatrists tend to feel uncomfortable about responding to journalists and media contributors or even mistrust them. As a consequence, they often decline their interview request.

To create new opportunities for improving media coverage of suicide, the Papageno Program developed a Mini Media Training (MMT) session specifically designed for mental health professionals. The MMT aims at raising psychiatrists’ awareness about the importance of communicating about suicide, at delivering accurate information about the WE and PE, at strengthening their capacity to respond to journalists in compliance with the WHO recommendations and at promoting reliable resources. The training session consists in a 10-min roleplay where psychiatrists are asked to answer the questions of a fiction journalist about a celebrity suicide. After this mock interview, the psychiatrists are offered a feedback based on their responses. As a positive reinforcer, answers in line with the WHO recommendations are pinpointed and likely positive consequences highlighted. The instructor then informs the participants about not-cited recommendations and provides advice about how to introduce them to journalists. Finally, psychiatrists receive a booklet containing the French version of the WHO recommendations.

The primary objective of the present study is to assess the effect of the MMT on psychiatrists’ ability to communicate about suicide in a way that helps journalists apply the WHO recommendations. We also studied the predictors of this ability and of its progression after the MMT.

## Materials and Methods

### Participants

Participants were French-speaking voluntary psychiatrists and residents in psychiatry recruited during eight French national psychiatry congresses that took place between June 2017 and December 2019. All participants provided their oral consent.

### Procedure

The participants were approached randomly during the psychiatry congresses (T0). They were informed orally and in writing about the study as part as the recruitment procedure. They were told that they were going to participate in a research aiming at evaluating the ability of psychiatrists to respond journalists about suicidal events. Then, participants were briefly introduced to the Papageno Program and were given an overview of the study process. They were then administered self-questionnaires about their sociodemographic characteristics, before receiving a face-to-face MMT. The instructor that led the mock interviews was also the main investigator of the study (KW). The MMT started with a short, standardized introduction providing some elements of context about the fictious suicide that motivated the interview. The instructor pretended he was a journalist working for a local newspaper. He explained that the body a famous French actor had just been discovered. He added that the actor had left a suicide note to his wife.

To test the extent to which the participant was able to spontaneously avoid talking about the specific case of the actor but rather provide general advice to guide the journalist, the questions were formulated to create three levels of directivity:

1. “What can you tell me about this suicide” (no directivity).
2. “What can you tell me about suicide in general” (low directivity).
3. “Do you have any advice for us to write our article” (strong directivity).

Between 1 and 3 months later (T1), the participants were called to carry on a second MMT by phone. They were informed of this recall at the time of their recruitment, at T0. They were explained that they would receive a text message or e-mail to schedule the appointment. The structure of the interview was exactly the same as in the first MMT. The scenario was analogous as respect to the type of event but changed in content. The instructor told the participant that a young singer who participated to a TV show was found dead in one location and surrounded by objects suggesting a death by suicide. The death was told to have occurred 3 days after the leak of a sextape involving the singer. The questions that the mock journalist asked were the same as at T0.

Both T0 and T1 were audio recorded.

### Outcomes

To assess the effect of the MMT, we developed a 33-items score of compliance with WHO recommendations (the WHOr score). Twenty-seven items of this grid consisted in direct operationalization of the WHO recommendations. The last six items assessed whether the participant informed the journalist about the WE and PE, avoided taking about the specific suicide case, and promoted reliable resources such as the consultation of the WHO recommendations (Table 1). The audio-records of the interviews were screened based on the 33 items of the WHOr score. One point was attributed for each cited item, and the total score was calculated by summing the points (unweighted WHOr score). To measure the ability of the psychiatrist to spontaneously deliver the pieces of advice, we also calculated a weighted WHOr score attributing three points when the items were spontaneously cited, two points when they were cited after a lowly directive question and one point when they were cited after a highly directive question. The total weighted WHOr score was calculated by summing the points, then dividing by three to bring the total score to 33, like the unweighted WHOr score. These scores also made it possible to take into account the degree of adherence of psychiatrists to WHO recommendations. The higher these scores, the more the psychiatrists adhered to the recommendations.

**TABLE 1:** Items of the WHOr score and the codebook defining each of them.

**Generalities**

1. Make yourself available to journalists: Score only when the participant makes himself available to the journalist for after the interview.
2. Encourage the consultation of the WHO recommendations: Score only when the participant specifically or explicitly mentions WHO recommendations.
3. Consult reliable scientific resources.
4. Inform about the Werther effect.
5. Inform about the Papageno effect.
6. Refuse to comment on the specific case: Rate when the participant does not give his opinion on the suicide mentioned, or does not take a position on it. Do not rate responses that make assumptions related to the suicide mentioned, or that describe the facts related to it.

**WHO recommendation 1: take the opportunity to educate the public about suicide**

7. Advise against presenting suicide as a consequence of a single cause: Score when the participant explicitly refers to the multifactorial nature of suicide, or when he describes in detail the process of the suicidal crisis by highlighting the chain of the different factors involved.
8. Encourage people to remember that suicide is often associated with psychiatric illness or substance use: Rate only those responses that clearly emphasize that psychiatric illness or substance use is frequently associated with suicide.
9. Encourage talking about suicide/dispel myths: Rate responses that prompt the journalist to talk about suicide in general, as well as responses that encourage journalists to de-stigmatize suicide.
10. Encourage the inclusion of elements suggesting that suicide is as a major public health problem: Rate responses that use arguments indicating that suicide is a public health issue. Do not rate responses that simply mention the frequent nature of suicide.
11. Encourage giving information about suicide risk factors or warning signs: Rate the responses that suggest the existence of risk factors for suicide by explicitly using the expression, or by mentioning risk factors by presenting them as such. Rate the responses that suggest the existence of warning signs at the onset of suicide, either explicitly using the expression or an equivalent, or by describing the dynamic process of the suicidal crisis.
12. Encourage mentioning the suicidal thoughts that preceded the act: Score only those responses that link the presence of suicidal ideation to a suicide act. Do not rate responses that only evoke suicidal ideation without associating them with a suicidal gesture.

**WHO recommendation 2: avoid any language that sensationalizes or normalizes suicide, or presents it as a solution**

13. Discourage language elements that tend to sensationalize, normalize, trivialize, or criminalize the act of suicide: Rate the responses that make the journalist aware of the importance of the elements of language used in talking about a suicide, or that encourage thejournalist to avoid any emotional content in the mention of a suicidal fact.
14. Encourage not to use expressions such as “successful suicide” or “failed attempt”.
15. Discourage language elements that contribute to presenting suicide as a solution: Rate only those responses that explicitly encourage the journalist not to present suicide as a solution.

**WHO recommendation 3: avoid prominent placement and undue repetition of stories about suicide**

16. Avoid repeating the coverage a suicide story/questioning the relevance of a new article: Rate the responses which insist on the harmful effect of the repeated media coverage of a suicidal event, or which explicitly question the relevance of writing an article on this subject. Do not rate responses that question the content of the article, without questioning the very existence of the article.
17. Recommend not placing the article on the first page or at the top of the page: Rate only those responses that clearly indicate that the article dealing with the suicide incident should not be featured in the newspaper.
18. In general, advise against highlighting and over-mediating suicidal events: Rate the responses that clearly emphasize the necessary differentiation that must be made, by the media, between the specific suicidal act, and suicide in general, and which clearly indicates that the emphasis should be on the second and not on the first.

**WHO recommendation 4: avoid explicit descriptions of the method used in a completed or attempted suicide**

19. Recommend not detailing the means used during a suicide or attempted suicide: Rate only those responses that clearly indicate that suicidal means should not be used in the article or story.

**WHO recommendation 5: avoid providing detailed information about the site of a completed or attempted suicide**

20. Advise against any detailed information concerning the place where the suicide or attempted suicide took place, as well as the history of this place in matters of suicide: Rate only those responses that clearly indicate that the location of the suicide took place in the article or story.
21. Advise against any details concerning the suicidal act: Rate only those responses that clearly advise reporters against giving details of the suicide incident itself. Do not rate responses that advise against dealing with suicide in particular without emphasizing not going into the details of the suicide in question.

**WHO recommendation 6: word headlines carefully**

22. Discourage the use of the word “suicide” in headlines, or the location or method of suicide.

**WHO recommendation 7: exercise caution in using photographs or video footage**

23. Discourage using inappropriate image(s) about the suicide.
24. Invite not to publish the content of the farewell letter: Score only those responses that clearly indicate that publication of the found farewell letter should be avoided.

**WHO recommendation 8: take particular care when reporting celebrity suicides**

25. Encourage the treatment of a celebrity suicide or attempted suicide with caution, without valuing the gesture and/or by recontextualizing: Rate only the responses that link the specificity of a celebrity’s suicide, with at least one of the following three points concerning the media coverage of this suicide: caution, lack of valuation of the gesture, and recontextualization (or an equivalent at least one of the three). Do not rate responses that simply evoke the celebrity of the person without making the connection to one of the three dimensions mentioned above.
26. Encourage to focus on the consequences that the suicidal behavior will have: Rate the answers that clearly mention one type of possible consequence of the act. Do not rate responses that simply state that there will be consequences, without specifying which ones.
27. Discourage speculation about the possibility of a suicidal cause for the unexplained death of a celebrity: Rate only those responses that stress the need to be certain that the fact mentioned is indeed suicide. Do not rate responses that imply that the suicide in question is certain.

**WHO recommendation 9: show due consideration for people bereaved by suicide**

28. Encourage respect for the privacy of the family and friends of the person affected by the suicide.
29. Recommend avoiding the interviews of families and friends persons who died by suicide.

**WHO recommendation 10: provide information about where to seek help**

30. Encourage giving information about resources/where to find help: Rate only those responses that clearly cite a specific worker or structure from which a person with suicidal thoughts can find help.
31. Encourage people to talk about the possibility of action, of care: Rate the responses that evoke the possibility of aid, prevention, care, and the fight against suicide. Do not rate the answers that start from the example of the deceased person to evoke the possibility of taking charge of a suicidal crisis.
32. Encourage giving examples of interventions that have contributed to prevent suicidal behaviors: Rate responses that suggest that the journalist include an insert in the article that includes the testimony of a person who has successfully overcome a suicidal crisis.

**WHO recommendation 11: recognize that media professionals themselves may be affected by stories about suicide**

33. Mention the potential impact and/or resonance that a suicidal behavior can induce in journalists: Rate only the answers clearly mentioning the resonances that the suicidal act can have on the journalist.

Ratings were carried out by two researchers (KW and SV, who practiced rating based on recordings collected during a pilot study at current work Kappa coefficients were calculated for each of the items (see Supplementary Table 1). The items for which the Kappa was less than 0.6 (*N* = 11) were reviewed by the 2 assessors for harmonization of the rating (Landis and Koch, 1977).

### Co-variables

We collected several variables to describe the sample: (1) socio-demographic characteristics: age (in years), gender (male, female), (2) professional information: status (psychiatrist, resident in psychiatry), professional experience (in years), hospital activity (yes, no), (3) level of knowledge (high if the participant reported to know the WE, the PE and the WHO recommendations, medium if the respondent declared to know one of the three notions, and low if none of the notions were known), and (4) media relations: previous experience with journalists (no experience, have already been solicited but without having answered, having already been interviewed).

### Analysis

The baseline characteristics were described and compared between respondents and non-respondents at T1 using Chi-square tests for proportions and Student’s tests for means.

To assess the effect of the MMT, analyses were only performed in participants who responded to both T0 and T1. Effect-sizes of change in mean WHOr scores were calculated using Cohen’s d for a paired samples by dividing the mean difference by the standard deviation of the difference. Within-subjects *t*-test was performed to compare WHOr scores at T0 and T1.

To identify factors likely to influence the WHOr score and its improvement after the MMT, we computed multivariate linear regression models with the WHOr scores at T0 and T1 being the outcome variable.

We adjusted the models for gender (male, female), professional experience (in years, categorized into four quartiles), hospital activity (yes, no), level of knowledge (high, medium, and low) and history regarding relations with media (no experience, solicited, and interviewed). Age and status were not included due to redundancy with the information provided by the variable “professional experience.” The models explaining the WHOr scores at T1 were additionally adjusted for the score at baseline. Associations between factors and WHOr scores were presented as differences and 95% confidence intervals (CI 95%).

The tests were two-tailed, and the level of significance was 0.05. Analyses were performed using R 3.6.1.

## Results

### Sample Characteristics

The characteristics of the respondents are described in Table 2. Among the psychiatrists and residents in psychiatry attending the French national psychiatry congresses, 173 volunteered to participate in the study at T0, and 132 also participated at T1 (response rate = 76%). No differences at baseline were found between respondents and non-respondents at T1.

**TABLE 2:** Baseline characteristics of the respondents and non-respondents at T1.

		Overall sample	Non-respondents at T1	Respondents at T1	*p*
		*N* = 173	*N* = 41	*N* = 132	
Age, *m* (sd)		41.7 (15.7)	45.5 (15.2)	40.5 (15.7)	0.074
Genre, *n* (%)					0.913
	Male	81 (46.8)	20 (48.8)	61 (46.2)	
	Female	92 (53.2)	21 (51.2)	71 (53.8)	
Status, *n* (%)					0.127
	Psychiatrist	116 (67.1)	32 (78.0)	84 (63.6)	
	Resident	57 (32.9)	9 (22.0)	48 (36.4)	
Professional experience, *m* (sd)		14.6 (14.1)	17.4 (14.1)	13.8 (14.1)	0.153
Hospital activity, *n* (%)					0.112
	Yes	132 (76.3)	27 (65.9)	105 (79.5)	
	No	41 (23.7)	14 (34.1)	27 (20.5)	
Previous experience with journalists, *n* (%)					0.224
	No previous experience	77 (44.5)	20 (48.8)	57 (43.2)	
	Solicited	16 (9.2)	1 (2.4)	15 (11.4)	
	Interviewed	80 (46.2)	20 (48.8)	60 (45.5)	
Previous knowledge, *n* (%)					0.590
	Low	20 (11.6)	19 (46.3)	54 (40.9)	
	Medium	80 (46.2)	19 (46.3)	61 (46.2)	
	High	73 (42.2)	3 (7.3)	17 (12.9)	
Unweighted WHOr score, *m* (sd)		4.64 (2.65)	4.39 (2.20)	4.72 (2.78)	0.489
Weighted WHOr score, *m* (sd)		2.97 (1.88)	2.81 (1.43)	3.02 (2.00)	0.535

### Impact of the Mini Media Training

**FIGURE 1:**
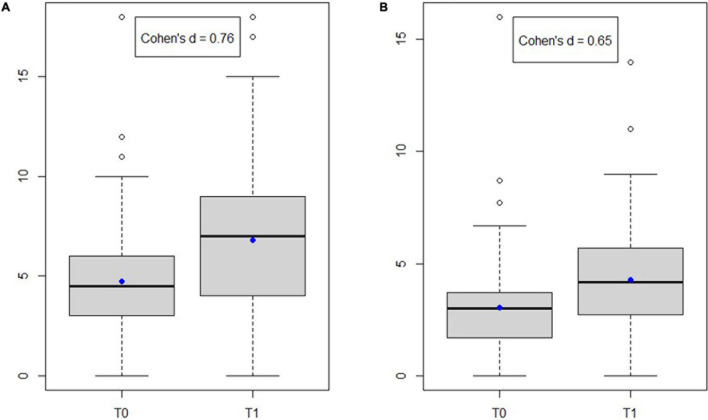
Unweighted **(A)** and weighted **(B)** WHOr score before (T0) and after (T1) the Mini Media Training. The mean score is symbolized by a blue dot on the boxplot.

Both the unweighted and the weighted WHOr scores increased at T1 (Figure 1). The average unweighted WHOr score was 4.72 (±2.78) at T0 and 6.80 (±3.41) at T1 (difference = 2.08, *p* < 0.001). The average weighted WHOr score was 3.02 (±2.00) at T0 and 4.26 (±2.31) at T1 (difference = 1.24, *p* < 0.001). Depending on whether we relied on the unweighted or weighted WHOr score, this corresponded to a Cohen’s d equal to 0.76 or 0.65, respectively.

The detailed responses to T0 and T1, item by item and depending on the level of directivity, are provided in Supplementary Table 2.

### Factors Associated With the WHOr Score

**FIGURE 2:**
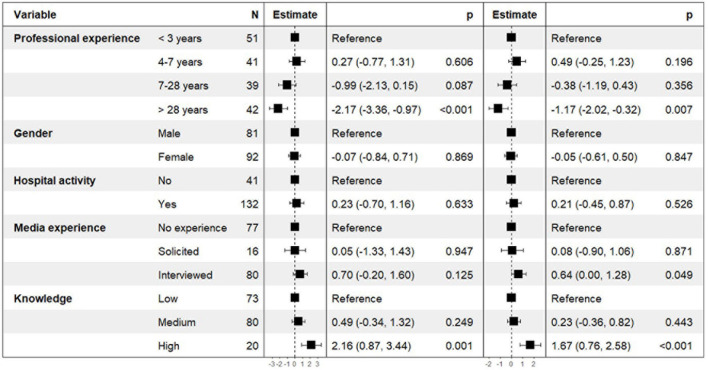
Factors associated with the unweighted and the weighted WHOr scores at T0–Results of the multivariate linear regression models.

Results of the models explaining the WHOr scores at T0 are presented in Figure 2. Compared to psychiatrists with short professional experience (<3 years), those reporting a long professional experience (>28 years) had a significantly lower WHOr score at T0 (estimate [CI 95%] = −2.17 [−3.36, −0.97], *p* < 0.001 for the unweighted WHOr score, and −1.17 [−2.02, −0.32], *p* = 0.007 for the weighted score. Conversely, a high level of knowledge was associated with a better WHOr score at T0 compared to a low level of knowledge (estimate [CI 95%] = 2.16 [0.87, 3.44], *p* = 0.001 for the unweighted WHOr score, and 1.67 [0.76, 2.58], *p* < 0.001 for the weighted WHOr score). In the model explaining the weighted WHOr score, having an experience of media interview was also associated with a score was also associated with a higher score (estimate [CI 95%] = 0.64 [0.01, 1.28], *p* = 0.049).

**FIGURE 3:**
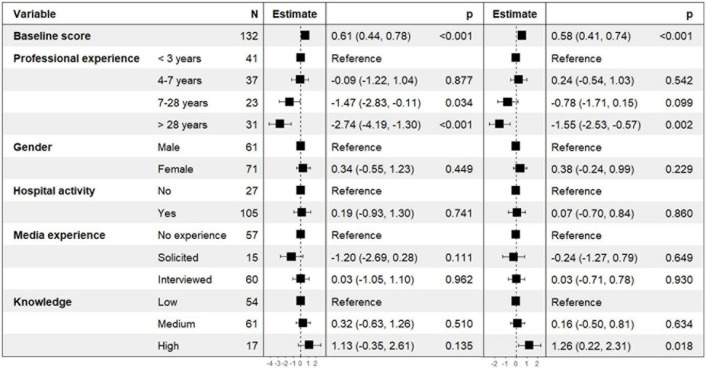
Factors associated with the unweighted and the weighted WHOr scores at T1–Results of the multivariate linear regression models.

Results of the models explaining the WHOr scores at T1 are presented in Figure 3. After adjusting for the score at baseline (the higher the T0 score, the higher the T1 score), professional experience was still associated with a lower WHOr score at T1. Compared to psychiatrists reporting an experience under 3 years, those with a more than 28 years of experience had a lower WHOr score at T1 (estimate [CI 95%] = −2.74 [−4.19, −1.30], *p* < 0.001 for the unweighted WHOr score, and −1.55 [−2.53, −0.57], *p* = 0.002 for the weighted WHOr score). In the model explaining the unweighted WHOr score, this difference was also significant for those reporting 7–28 years of experience (estimate [CI 95%] = −1.47 [−2.83, −0.11], *p* < 0.034). A high level of knowledge was only associated with the weighted WHOr score (estimate [CI 95%] = 1.26 [0.22, 2.31], *p* = 0.018).

## Discussion

### Main Results

Among the 132 psychiatry physicians that completed both T0 and T1 assessments, the MMT resulted in significant improvements of both weighted and unweighted WHOr scores, suggesting better abilities in advising journalists about media coverage of suicide. Having a shorter professional experience, a history of contact with journalists and stronger general knowledge about the WE, PE, and the WHO recommendations were associated with greater communication skills at baseline. All these characteristics, along with prior experience in communicating with media, also predicted a better weighted WHOr score at T1.

### Interpretation

These results support the relevance of the MMT for improving psychiatrists’ ability to guide journalists toward more responsible media coverage of suicide. After the MMT, physicians where more prone to deliver relevant pieces of advice in relation with the WHO recommendations, as suggested by the significant T0–T1 increase of the WHOr unweighted score. The T0–T1 progression of the weighted score also indicate a stronger tendency to deliver these recommendations spontaneously. It is noteworthy–and maybe counterintuitive–that younger professionals have been found to be more comfortable communicating with journalists than their older counterparts. This may reflect a generational gap according to which younger populations are more aware of contemporaneous communication challenges because they grew in a highly mediatic environment (Horst, 2012). The consequences of communication about suicide, in particular, has gained a considerable interest in the past 10 years (Niederkrotenthaler and Till, 2019), with possible translations into the curriculum of young doctors. Also, minimal professional experience may be associated with greater plasticity of professional identity, and possibly greater availably to the acquisition of new professional skills. This may explain that the MMT appeared more beneficial to early career than to confirmed psychiatrists.

The participants had better performances during the interviews when they had greater prior knowledge of the WE, PE, and WHO recommendations. This result was expected since literacy is usually considered as a prerequisite for, or potentiator of, professional skills, especially as regards to suicide prevention (Inman et al., 1984). Although they did not necessarily know each of the recommendations, it is likely that psychiatrists relied on their general awareness of the WE and PE to infer the relevant pieces of advice to provide to the journalists. The level of prior knowledge about the effect of media coverage of suicide at baseline predicted a greater progression of the WHOr weighted score between T0 and T1, but not of the unweighted score. Rather than informing about the new pieces of advice to deliver, the MMT may have helped psychiatrists who already had knowledge about suicide-related communication to take a stronger lead during the interview and guide more proactively the journalists.

Finally, it was also expected that participants who already had contacts with journalists would have greater weighted scores at baseline than the rest of the sample. Indeed, already having dealt with journalists is likely to be associated with greater confidence during interviews, and therefore with greater ease in spontaneously giving advice to media professionals.

### Perspective

The journalistic work is driven by strong missions and constrains that might sometimes contradict prevention perspectives, especially when it comes to suicidal behaviors. For instance, the duty to provide information in a context of increasing economic pressure and of competition with social media incite media professionals to rapidly release high impact productions (Collings and Kemp, 2010). This may lead to structural contradictions with the principles of the WHO recommendations by urging journalists to use catchy headlines, explicit pictures or a sensational tone. Some journalists may also perceive externally applied recommendations as restrictions for their freedom of information (Notredame et al., 2016; Yaqub et al., 2020). Despite these possible sticking points, most media professionals show a strong interest in mental health issues. In a survey that inquired 20 journalists by a self-questionnaire sent over the Internet, 90% of the sample agreed that the press could be used as a vehicle for spreading information about mental health (Barasino et al., 2016; Legrand, 2016). However, the respondents also expressed some frustration because many psychiatrists refused, ignored, or responded after a long delay to their interview request. As a short, easy to implement pedagogical format, the MMT may offer a solution to mitigate this interprofessional gap. It encourages and give psychiatrists the tools to actively present themselves as resources for journalists. While breaking with a prescriptive or moralizing attitude, it helps them endorsing the role of prevention ambassadors, overcoming the possible points of tension between media demands and health principles, and, in turn, raise awareness in journalists. Beyond simply delivering technical advice, this effort to make media and mental health perspectives converge may be the condition for both mental health professionals and journalists to really grasp the challenges of the WHO recommendations and thus apply them (Notredame et al., 2016).

According to our results, students and young psychiatrists could be a primary target for such educative programs. Assuming that residency and early carrier is the privileged period for physicians to build their professional identity, delivering media-oriented training sessions such as the MMT during this time of the curriculum could help raising a culture of collaboration between journalists and mental health professionals at the benefit of suicide prevention (Notredame et al., 2016).

### Limits

To our knowledge, the present study is the first evaluated initiative taken to improve the communication skills of psychiatrists about suicide. However, several methodological limitations need to be taken in account. (1) The experimental setting was not exactly the same at T0 and T1. For ecological reasons, we organized a face-to-face simulation exercise at T0, while the interview was carried out by telephone at T1. This may alter the possibility to impute the observed score variations to the MMT because of reduced comparability between the assessment points. However, it is very unlikely that phone contacts had, by themselves, improved the performance of the psychiatrists. In addition, this protocol helped considerably minimizing the attrition bias which was less than 25%. (2) Due to agenda constrains of the participants, the time lag between T0 and T1 varied from 1 and 3 months, which may have introduced variability in the performances due to a memory retention bias. If our results allow to be pretty confident about the effect of the MMT at 1 month, other studies could be led to confirm the remanence of this effect on longer periods. (3) During the training interviews, the journalist adopted a neutral position without bouncing on the psychiatrist’s responses or pushing him to his limits. Such conditions may lack realism, and it is possible that the trained psychiatrists would show poorer performances in an ecological context. An interesting development path for MMT could be to divide the participants into two groups, one with a journalist adopting a neutral tone, the other with a journalist with a more aggressive style. Such work would make it possible to better understand the potential influence of journalistic style on the quality of the psychiatrist’s responses. (4) Investigators had to deal with rating ambiguities between some items of the WHOr scale. For example, the items “Avoid repeating the coverage of a suicidal behavior/questioning the relevance of a new article” and “In general, advise against highlighting and over-mediating suicidal events” were sometimes overlapping (5) As the MMT evaluates the effectiveness of the entire intervention, i.e., the debriefing associated with the brochure given to the participant at the end of T0, it was unfortunately not possible to attribute the observed effect to any of the components of MMT. (6) Although still very influent in France and worldwide (Newman et al., 2021), journalism isn’t the only source of suicide-related media productions. For instance, suicidal contents are frequent on websites (Baker and Fortune, 2008) and social media (Ruder et al., 2011), with a large panel of stakeholders involved such as citizens, webmasters, or blog moderators. Also, the notion of “suicidal contents” cannot be summed up to reports of suicidal behaviors. Testimonies, general information, posts or fictions may also be concerned by the risk of WE and opportunity of PE. To that respect the MMT has a rather narrow scope and specific variations should be considered.

## Conclusion

The MMT appears as an interesting tool to improve psychiatrists’ ability to guide journalists toward a more responsible media coverage of suicide. In a relatively short time, it raises awareness about the effects of suicide-related communication and trains the participants to concretely help journalists better applying the WHO recommendations. Because actioning indirect levers, its sole implementation would certainly not be sufficient to reduce the WE and promote the PE. However, the MMT aims at involving psychiatrists as active stakeholders in the collective effort for safer media productions about suicide. As such, it may be synergistic with other actions organized in France by the Papageno program, such as the publication of national adaptation of the recommendations (Legrand, 2016), more complete media trainings (Legrand, 2016) or collaborations between mental health trainees and students in journalism (Notredame et al., 2016).

## Data Availability Statement

The raw data supporting the conclusions of this article will be made available by the authors, without undue reservation.

## Ethics Statement

Ethical review and approval was not required for the study on human participants in accordance with the local legislation and institutional requirements. Written informed consent for participation was not required for this study in accordance with the national legislation and the institutional requirements. Written informed consent was not obtained from the individual(s) for the publication of any potentially identifiable images or data included in this article.

## Author Contributions

C-EN and NP were responsible for the original idea of the Mini-Media Training. C-EN conceived the protocol. KW carried out the Mini-Media Training sessions and collected the data. KW and SV rated the interviews and managed the data. MW performed the analysis. KW, C-EN, and MW wrote the manuscript with support from NP, SV, PG, and GV. GV supervised the whole project. All authors contributed to the article and approved the submitted version.

## Conflict of Interest

The authors declare that the research was conducted in the absence of any commercial or financial relationships that could be construed as a potential conflict of interest.

## Publisher’s Note

All claims expressed in this article are solely those of the authors and do not necessarily represent those of their affiliated organizations, or those of the publisher, the editors and the reviewers. Any product that may be evaluated in this article, or claim that may be made by its manufacturer, is not guaranteed or endorsed by the publisher.

## Footnotes

1
www.papageno-suicide.com

## Supplementary Material

Supplementary Table 1 Initial kappa coefficients for each item (in red, items whose initial kappa coefficients were less than 0.6).

Supplementary Table 2 Responses to each of the items of the WHOr score according to the level of directivity at T0 and T1 (paired sample, *N* = 132).

## Abbreviations

MMT: Mini Media Training
PE: Papageno Effect
WE: Werther Effect
WHO: World Health Organization.
