# Supplementary material for: A Short Media Training Session Is Effective in Reinforcing Psychiatrists’ Communication Skills About Suicide
Source: Front Psychol. 2021 Sep 16;12:733691. doi: 10.3389/fpsyg.2021.733691 (PMC8481656; doi:10.3389/fpsyg.2021.733691)
Supplement: Supplementary Table 1 — Initial kappa coefficients for each item (in red, items whose initial kappa coefficients were less than 0.6). [file Table_1.docx]

Supplementary table 1: Initial kappa coefficients for each item (in red, items whose initial kappa coefficients were less than 0.6)

| **Item** | **Initial Kappa Coefficient** |
| --- | --- |
| 1. Make yourself available to journalists | 0,43 |
| 1. Encourage the consultation of the WHO recommendations | 0,92 |
| 1. Consult reliable scientific resources | 0,64 |
| 1. Inform about the Werther effect | 0,79 |
| 1. Inform about the Papageno effect | 0,56 |
| 1. Refuse to comment on the specific case | 0,60 |
| 1. Advise against presenting suicide as a consequence of a single cause | 0,64 |
| 1. Encourage people to remember that suicide is often associated with psychiatric illness or substance use | 0,65 |
| 1. Encourage talking about suicide / dispel myths | 0,34 |
| 1. Encourage the inclusion of elements suggesting that suicide is as a major public health problem | 0,62 |
| 1. Encourage giving information about suicide risk factors or warning signs | 0,55 |
| 1. Encourage mentioning the suicidal thoughts that preceded the act | 0,22 |
| 1. Discourage language elements that tend to sensationalize, normalize, trivialize or criminalize the act of suicide | 0,62 |
| 1. Encourage not to use expressions such as “successful suicide” or “failed attempt” | 0,67 |
| 1. Discourage language elements that contribute to presenting suicide as a solution | 0,14 |
| 1. Avoid repeating the coverage a suicide story / questioning the relevance of a new article | 0,67 |
| 1. Recommend not placing the article on the first page or at the top of the page | 0,87 |
| 1. In general, advise against highlighting and over-mediating suicidal events | 0,33 |
| 1. Recommend not detailing the means used during a suicide or attempted suicide | 0,81 |
| 1. Advise against any detailed information concerning the place where the suicide or attempted suicide took place, as well as the history of this place in matters of suicide | 0,92 |
| 1. Advise against any details concerning the suicidal act | 0,69 |
| 1. Discourage the use of the word “suicide” in headlines, or the location or method of suicide | 0,83 |
| 1. Discourage using inappropriate image(s) about the suicide | 0,87 |
| 1. Invite not to publish the content of the farewell letter | 1 |
| 1. Encourage the treatment of a celebrity suicide or attempted suicide with caution, without valuing the gesture and / or by recontextualizing | 0,36 |
| 1. Encourage to focus on the consequences that the suicidal behavior will have | 0,33 |
| 1. Discourage speculation about the possibility of a suicidal cause for the unexplained death of a celebrity | 0,69 |
| 1. Encourage respect for the privacy of the family and friends of the person affected by the suicide | 0,67 |
| 1. Recommend avoiding the interviews of families and friends persons who died by suicide | 0 |
| 1. Encourage giving information about resources / where to find help | 0,67 |
| 1. Encourage people to talk about the possibility of action, of care | 0,50 |
| 1. Encourage giving examples of interventions that have contributed to prevent suicidal behaviors | 0,78 |
| 1. Mention the potential impact and / or resonance that a suicidal behavior can induce in journalists | 1 |
