# Supplementary material for: A Short Media Training Session Is Effective in Reinforcing Psychiatrists’ Communication Skills About Suicide
Source: Front Psychol. 2021 Sep 16;12:733691. doi: 10.3389/fpsyg.2021.733691 (PMC8481656; doi:10.3389/fpsyg.2021.733691)
Supplement: Supplementary Table 2 — Responses to each of the items of the WHOr score according to the level of directivity at T0 and T1 (paired sample, N = 132). [file Table_2.docx]

Supplementary table 2: Responses to each of the items of the WHOr score according to the level of directivity at T0 and T1 (paired sample, N = 132)

|  |  | T0 (N = 132) | | T1 (N = 132) | |  |
| --- | --- | --- | --- | --- | --- | --- |
| Item | Directivity | N | (%) | N | (%) | p |
| 1 | No | 2 | (1.5) | 1 | (0.8) | 1,000 |
| 1 | Low | 0 | (0.0) | 1 | (0.8) | 1,000 |
| 1 | Strong | 2 | (1.5) | 2 | (1.5) | 1,000 |
| 2 | No | 1 | (0.8) | 2 | (1.5) | 1,000 |
| 2 | Low | 0 | (0.0) | 0 | (0.0) | 1,000 |
| **2** | **Strong** | **3** | **(2.3)** | **14** | **(10.6)** | **0.01** |
| 3 | No | 1 | (0.8) | 2 | (1.5) | 1,000 |
| 3 | Low | 0 | (0.0) | 0 | (0.0) | 1,000 |
| 3 | Strong | 11 | (8.3) | 7 | (5.3) | 0.343 |
| **4** | **No** | **2** | **(1.5)** | **14** | **(10.6)** | **0.003** |
| 4 | Low | 0 | (0.0) | 3 | (2.3) | 0.248 |
| 4 | Strong | 21 | (15.9) | 34 | (25.8) | 0.061 |
| 5 | No | 0 | (0.0) | 2 | (1.5) | 0.479 |
| 5 | Low | 1 | (0.8) | 0 | (0.0) | 1,000 |
| 5 | Strong | 3 | (2.3) | 6 | (4.5) | 0.505 |
| 6 | No | 61 | (46.2) | 73 | (55.3) | 0.082 |
| 6 | Low | 0 | (0.0) | 0 | (0.0) | 1,000 |
| 6 | Strong | 0 | (0.0) | 0 | (0.0) | 1,000 |
| 7 | No | 13 | (9.8) | 18 | (13.6) | 0.332 |
| 7 | Low | 6 | (4.5) | 8 | (6.1) | 0.773 |
| 7 | Strong | 9 | (6.8) | 8 | (6.1) | 1,000 |
| 8 | No | 13 | (9.8) | 10 | (7.6) | 0.628 |
| 8 | Low | 39 | (29.5) | 30 | (22.7) | 0.200 |
| 8 | Strong | 4 | (3.0) | 3 | (2.3) | 1,000 |
| 9 | No | 4 | (3.0) | 6 | (4.5) | 0.752 |
| 9 | Low | 12 | (9.1) | 14 | (10.6) | 0.814 |
| 9 | Strong | 7 | (5.3) | 9 | (6.8) | 0.803 |
| 10 | No | 16 | (12.1) | 12 | (9.1) | 0.502 |
| 10 | Low | 45 | (34.1) | 47 | (35.6) | 0.868 |
| 10 | Strong | 3 | (2.3) | 2 | (1.5) | 1,000 |
| 11 | No | 12 | (9.1) | 6 | (4.5) | 0.181 |
| **11** | **Low** | **21** | **(15.9)** | **37** | **(28.0)** | **0.008** |
| 11 | Strong | 6 | (4.5) | 2 | (1.5) | 0.221 |
| 12 | No | 0 | (0.0) | 1 | (0.8) | 1,000 |
| 12 | Low | 4 | (3.0) | 1 | (0.8) | 0.248 |
| 12 | Strong | 0 | (0.0) | 0 | (0.0) | 1,000 |
| **13** | **No** | **3** | **(2.3)** | **12** | **(9.1)** | **0.026** |
| 13 | Low | 2 | (1.5) | 3 | (2.3) | 1,000 |
| 13 | Strong | 39 | (29.5) | 46 | (34.8) | 0.349 |
| 14 | No | 1 | (0.8) | 0 | (0.0) | 1,000 |
| 14 | Low | 3 | (2.3) | 0 | (0.0) | 0.248 |
| 14 | Strong | 1 | (0.8) | 1 | (0.8) | 1,000 |
| 15 | No | 0 | (0.0) | 0 | (0.0) | 1,000 |
| 15 | Low | 2 | (1.5) | 2 | (1.5) | 1,000 |
| 15 | Strong | 0 | (0.0) | 3 | (2.3) | 0.248 |
| 16 | No | 0 | (0.0) | 0 | (0.0) | 1,000 |
| 16 | Low | 0 | (0.0) | 0 | (0.0) | 1,000 |
| 16 | Strong | 1 | (0.8) | 6 | (4.5) | 0.131 |
| 17 | No | 0 | (0.0) | 0 | (0.0) | 1,000 |
| 17 | Low | 0 | (0.0) | 0 | (0.0) | 1,000 |
| **17** | **Strong** | **0** | **(0.0)** | **12** | **(9.1)** | **0.001** |
| 18 | No | 1 | (0.8) | 2 | (1.5) | 1,000 |
| 18 | Low | 0 | (0.0) | 1 | (0.8) | 1,000 |
| 18 | Strong | 5 | (3.8) | 3 | (2.3) | 0.724 |
| 19 | No | 4 | (3.0) | 7 | (5.3) | 0.371 |
| 19 | Low | 0 | (0.0) | 0 | (0.0) | 1,000 |
| **19** | **Strong** | **14** | **(10.6)** | **39** | **(29.5)** | **<0.001** |
| 20 | No | 0 | (0.0) | 3 | (2.3) | 0.248 |
| 20 | Low | 0 | (0.0) | 0 | (0.0) | 1,000 |
| **20** | **Strong** | **3** | **(2.3)** | **15** | **(11.4)** | **0.003** |
| 21 | No | 3 | (2.3) | 4 | (3.0) | 1,000 |
| 21 | Low | 0 | (0.0) | 1 | (0.8) | 1,000 |
| **21** | **Strong** | **13** | **(9.8)** | **36** | **(27.3)** | **<0.001** |
| 22 | No | 1 | (0.8) | 0 | (0.0) | 1,000 |
| 22 | Low | 0 | (0.0) | 1 | (0.8) | 1,000 |
| 22 | Strong | 1 | (0.8) | 6 | (4.5) | 0.131 |
| 23 | No | 1 | (0.8) | 1 | (0.8) | 1,000 |
| 23 | Low | 0 | (0.0) | 0 | (0.0) | 1,000 |
| **23** | **Strong** | **0** | **(0.0)** | **7** | **(5.3)** | **0.023** |
| 24 | No | 1 | (0.8) | 0 | (0.0) | 1,000 |
| 24 | Low | 0 | (0.0) | 0 | (0.0) | 1,000 |
| 24 | Strong | 2 | (1.5) | 1 | (0.8) | 1,000 |
| **25** | **No** | **1** | **(0.8)** | **11** | **(8.3)** | **0.009** |
| 25 | Low | 0 | (0.0) | 3 | (2.3) | 0.248 |
| 25 | Strong | 4 | (3.0) | 10 | (7.6) | 0.181 |
| **26** | **No** | **13** | **(9.8)** | **28** | **(21.2)** | **0.012** |
| 26 | Low | 3 | (2.3) | 8 | (6.1) | 0.182 |
| 26 | Strong | 13 | (9.8) | 16 | (12.1) | 0.628 |
| 27 | No | 5 | (3.8) | 13 | (9.8) | 0.061 |
| 27 | Low | 0 | (0.0) | 0 | (0.0) | 1,000 |
| 27 | Strong | 3 | (2.3) | 2 | (1.5) | 1,000 |
| 28 | No | 5 | (3.8) | 3 | (2.3) | 0.724 |
| 28 | Low | 0 | (0.0) | 1 | (0.8) | 1,000 |
| 28 | Strong | 12 | (9.1) | 15 | (11.4) | 0.628 |
| 29 | No | 0 | (0.0) | 0 | (0.0) | 1,000 |
| 29 | Low | 0 | (0.0) | 0 | (0.0) | 1,000 |
| 29 | Strong | 0 | (0.0) | 2 | (1.5) | 0.479 |
| 30 | No | 3 | (2.3) | 11 | (8.3) | 0.061 |
| 30 | Low | 20 | (15.2) | 27 | (20.5) | 0.296 |
| 30 | Strong | 28 | (21.2) | 41 | (31.1) | 0.055 |
| **31** | **No** | **16** | **(12.1)** | **29** | **(22.0)** | **0.037** |
| 31 | Low | 47 | (35.6) | 58 | (43.9) | 0.208 |
| 31 | Strong | 22 | (16.7) | 19 | (14.4) | 0.719 |
| 32 | No | 0 | (0.0) | 0 | (0.0) | 1,000 |
| 32 | Low | 1 | (0.8) | 1 | (0.8) | 1,000 |
| **32** | **Strong** | **0** | **(0.0)** | **12** | **(9.1)** | **0.001** |
| 33 | No | 0 | (0.0) | 0 | (0.0) | 1,000 |
| 33 | Low | 0 | (0.0) | 0 | (0.0) | 1,000 |
| 33 | Strong | 1 | (0.8) | 1 | (0.8) | 1,000 |
